# Prospective Study of Arterial Stiffness and Subsequent Cognitive Decline Among Community-Dwelling Older Japanese

**DOI:** 10.2188/jea.JE20140250

**Published:** 2015-09-05

**Authors:** Yu Taniguchi, Yoshinori Fujiwara, Yu Nofuji, Mariko Nishi, Hiroshi Murayama, Satoshi Seino, Rika Tajima, Yutaka Matsuyama, Shoji Shinkai

**Affiliations:** 1 Research Team for Social Participation and Community Health, Tokyo Metropolitan Institute of Gerontology, Tokyo, Japan; 2 Department of Biostatistics, School of Public Health, University of Tokyo, Tokyo, Japan

**Keywords:** cognitive decline, arterial stiffness, older persons

## Abstract

**Background:**

Brachial-ankle pulse wave velocity (baPWV) is inversely associated with cognitive function. However, it is not known whether baPWV predicts cognitive decline (CD) in later life. We examined whether or not baPWV is an independent risk marker of subsequent CD in a population of older Japanese.

**Methods:**

Among 982 adults aged 65 years or older who participated in a baseline survey, 526 cognitively intact adults (Mini-Mental State Examination [MMSE] score ≥24; mean [SD] age, 71.7 [5.6] years; women, 57.8%) were followed for a period of up to 5 years. Pulse wave velocity was determined using an automated waveform analyser. Cognition was assessed by the MMSE, and CD was defined as a decrease of two points or more on the MMSE.

**Results:**

During an average follow-up of 3.4 years, 85 participants (16.2%) developed CD. After controlling for important confounders, the odds ratios for CD in the highest and middle tertiles of baPWV, as compared with the lowest tertile, were 2.95 (95% confidence interval, 1.29–6.74) and 2.39 (95% confidence interval, 1.11–5.15), respectively.

**Conclusions:**

High baPWV was an independent predictor of CD in a general population of older adults and may be useful in the clinical evaluation of elders.

## INTRODUCTION

Dementia is a growing public health concern worldwide. The World Health Organization estimates that the number of people with dementia will double by 2030 and more than triple by 2050.^1^ Dementia is a major cause of disability and need for premature care in older adults. It also affects the wellbeing of caregivers, who experience physical and mental stress during care, and is an economic burden due to the costs associated with providing social care. Alzheimer’s disease (AD) causes long-term pathological changes, and previous studies noted an association between the prodromal stage of AD and cognitive decline (CD). Wilson and colleagues^2^ reported that, compared with a group with no cognitive impairment, the annual rate of CD was more than doubled in a group with mild cognitive impairment and more than quadrupled in a group with AD. There is no established treatment for dementia; however, prevention or delay of CD might assist in limiting the onset of future dementia.

We previously reported several independent risk markers in CD incidence, including lower physical performance^3^ and nutritional biomarkers.^4^ Moreover, arterial stiffness (loss of cushioning capacity) was recently used as risk factor for CD in epidemiological and clinical studies. Arterial stiffness is associated with physical activity and nutritional status, is a marker of functional and structural changes in arteries, and can be assessed noninvasively using pulse wave velocity (PWV) measurement.^5^ Previous studies used carotid-femoral PWV (cfPWV), which measures the velocity of the pulse wave as it travels a given distance between the carotid and femoral arteries^6^ and reflects the stiffness of elastic arteries.^7^ A high cfPWV was significantly associated with marked declines in psychomotor speed,^8^ verbal learning, and nonverbal memory^9^ in older adults. Scuteri and colleagues^10^^,^^11^ reported an association between cfPWV and CD among older adults who reported memory problems and were assessed using the Mini-Mental State Examination (MMSE). However, no independent association was found between cfPWV and CD in the Rotterdam Study.^12^

Although cfPWV is regarded throughout the world as the gold standard for measurement of arterial stiffness, brachial-ankle PWV (baPWV) measurement is more frequently used in Japan.^7^ This method was developed to assess pulse wave transmission between the brachial and tibial arteries^13^ and reflects stiffness in both muscular and elastic arteries.^7^ The baPWV technique has good reproducibility, even when not performed by highly skilled technicians, and is not time-consuming. Earlier cross-sectional studies found that baPWV was inversely associated with cognitive function among Japanese aged 70 years or older^14^ and 85 years.^5^ However, the longitudinal association between baPWV and CD has not been investigated among community-dwelling elderly individuals. Furthermore, whether or not arterial stiffness is associated with CD after adjustment for sociomedical factors is unknown.

A better understanding of the relation of arterial stiffness to CD risk might yield new insights regarding physiological mechanisms during the prodromal stage of dementia. In this prospective study of community-dwelling older adults, we measured baPWV and health-related factors, including measures of physical performance, nutritional biomarkers, chronic diseases, and apolipoprotein E (APOE) genotype, at baseline. We then used the MMSE to follow the cognitive function of participants. The objective of the study was to determine whether baPWV was independently associated with CD after adjustment for potential confounders.

## METHODS

### Participants

**Figure 1.**
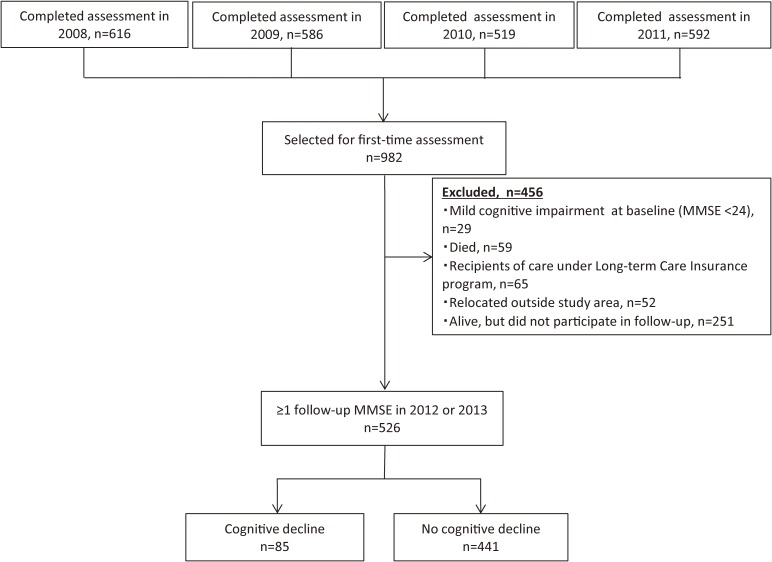
Study flow according to cognitive outcome.

Data were collected as part of a comprehensive health examination conducted in Kusatsu Town, Gunma Prefecture, Japan. In addition to an annual preventive health check-up, for residents aged 40 years or older, participants aged 65 years or older underwent a geriatric assessment that included measurement of baPWV and cognitive function. The details of the study design have been previously reported.^3^^,^^4^ All participants provided written informed consent under conditions approved by the Ethics Committee at Tokyo Metropolitan Institute of Gerontology. Baseline assessments were performed from 2008 through 2011 at the same local public health center. Annual follow-up assessments were conducted in the same manner from 2012 through 2013. To be eligible for the study, individuals had to complete the baseline assessment at least once. Among the 2313 elders with valid and complete baseline data, 982 were selected for participation in the present study. Ultimately, data from 526 adults who had completed both baseline and follow-up assessments were analyzed, as shown in Figure 1. The reasons for attrition during the follow-up period (*n* = 456) were operationally defined as mild cognitive impairment at baseline (MMSE score <24; *n* = 29), death (*n* = 59), need for care under the Long-term Care Insurance program (*n* = 65), relocation (*n* = 52), and unknown reasons (*n* = 251).

### Brachial-ankle pulse wave velocity

The baPWV (cm/sec) was measured with an automatic waveform analyzer (BP-203 RPE III; Omron Colin Co., Ltd., Tokyo, Japan). This technique has been described in detail elsewhere.^14^^–^^18^ Briefly, cuffs wrapped around the brachia and ankles were connected to a plethysmographic sensor, which determined the volume pulse form, and an oscillometric pressure sensor. Pressure waveforms were recorded simultaneously at the brachial and tibial arteries to determine the time interval between the initial rise in the brachial and tibial waveforms. The path length from the suprasternal notch to the elbow (*Δ*Da) was obtained from superficial measurements and was estimated using the equation *Δ*Da = 0.2195 × H − 2.0734, where H is the height of the participant in centimeters. The path length from the suprasternal notch to the ankle (*Δ*Db) was calculated as *Δ*Db = (0.5643 × H − 18.3810) + (0.2486 × H − 30.7090). The baPWV was calculated as the mean of the values from the left and right sides, and the data were divided into tertiles (≤1591, 1591–1889, and ≥1889 cm/s) in the analysis.

### Cognitive function

Cognitive function was assessed using the MMSE, which was administered by well-trained personnel.^3^^,^^4^ The score ranges from 0 to 30, with lower scores indicating poorer global cognitive function. A decrease of at least two points on the MMSE during the follow-up period was defined as CD.^19^^–^^21^

### Laboratory data

Blood testing included white blood cell (WBC) count, red blood cell count, hemoglobin, hematocrit, mean corpuscular volume, hemoglobin A1c, total cholesterol, high-density lipoprotein cholesterol, triglycerides, creatinine, and albumin. Nonfasting blood samples were collected using standard procedures. Samples were analyzed at the Sannaikai Clinic, which is regularly monitored by several domestic authorities. APOE genotyping was performed on coded DNA specimens by technicians blinded to the diagnosis. The categorical variable APOE was classified by genotype as ε2ε2, ε2ε3, ε2ε4, ε3ε3, ε3ε4, and ε4ε4.^22^

### Other variables

The covariates included sex, age, years of education, living arrangement, frequency of going outdoors, history of chronic diseases and antihypertensive drug use,^23^ body height and weight, body mass index (BMI), resting blood pressure, ankle-brachial pressure index (ABI), grip strength, usual and maximum gait speeds, Tokyo Metropolitan Institute of Gerontology Index of Competence (TMIG-IC), Geriatric Depression Scale (GDS) Short-version, and years of follow-up. Well-trained personnel interviewed the participants.

Chronic diseases included clinically relevant medical conditions: hypertension, hyperlipidemia, cerebral vascular disease (stroke, cerebral hemorrhage, and subarachnoid hemorrhage), heart disease (angina, myocardial infarction, arrhythmia, and others), and diabetes mellitus. For each condition, participants were asked whether a physician had diagnosed the specific condition (yes or no).

Grip strength (kg) was measured in the dominant hand using a standard hydraulic handgrip dynamometer^24^; participants were asked to squeeze the handle as hard as they could. Gait speed was measured over a straight 11-m walkway marked with tape at 3 m and 8 m.^25^^,^^26^ Participants were asked to walk at their usual and maximum pace, and well-trained staff measured the time required to walk 5 m and calculated gait speed (expressed as m/sec). Usual gait performance was measured once. Grip strength and maximum gait speed were measured twice, and the better of the two results was recorded.

The TMIG-IC^27^ was designed to measure higher-level competence in older community residents. The score ranges from 0 to 13, with higher scores indicating higher functional capacity.

### Statistical analyses

The Mann-Whitney *U* test and chi-square test were used to compare baseline sociomedical characteristics between individuals who developed CD during follow-up and those who did not. We used logistic regression plot to predict the probability of occurrence for subsequent CD with baPWV adjusted for sex, age, and follow-up year. The dataset for the present study does not include censored data, and the effect of baPWV at baseline on subsequent CD would not change during the follow-up period. Thus, we used multiple logistic regression models to examine independent associations between measures of baPWV at baseline with subsequent CD. We adjusted for confounding factors using multiple logistic regression models in which baPWV was defined as the independent variable, and subsequent CD was defined as the dependent variable. Some continuous variables were divided into tertiles as a covariate.

Four models were used. The first was the crude model (model 1). In the second, the covariates were sex, age, and follow-up year (model 2). Model 3 included the covariates in model 2 plus all factors that were significantly associated with CD in univariate analysis. In model 4, antihypertensive medication, systolic blood pressure, high-density lipoprotein cholesterol, albumin, and APOE genotype were added as important covariates. We excluded some factors, to avoid multicollinearity among covariates. The statistical models were run separately. Statistics were computed using SPSS (version 18.0; SPSS, Inc., Chicago, IL, USA) and SAS (version 9.4; SAS Institute, Inc., Cary, NC, USA), and the level of significance was set at *P* < 0.05.

## RESULTS

Among study participants at baseline, average (standard deviation [SD]) age was 71.7 (5.6) years, 57.8% were women, 22.5% lived alone, 13.2% had 13 or more years of education, 54.8% had maximum scores on the TMIG-IC, and 87.3% had a score of 26 or higher on the MMSE. Chronic diseases included clinically relevant medical conditions; 36.3% had hypertension (33.5% used antihypertensive drugs), 21.5% had hyperlipidemia, 4.0% had cerebral vascular disease, 11.4% had heart disease, and 10.3% had diabetes. The average (SD) baPWV (cm/sec) was 1782 (362).

During a mean follow-up of 3.4 years, 85 of 526 (16.2%) adults developed CD. Table 1 shows the baseline demographic and health characteristics of individuals who did and did not develop CD during the follow-up period. At baseline, participants who developed CD were older, had fewer years of education, were less likely to go outdoors, had lower usual and maximum gait speeds, had higher WBC counts, had higher MMSE scores, and had longer duration of follow-up compared to participants who did not develop CD; all of these variables were included as potential confounders in multivariate analysis.

**Table 1.** Baseline demographic and health characteristics of 526 community-dwelling Japanese aged ≥65 years with and without cognitive decline

Variable	Incident event (ΔMMSE ≤−2)		

	Cognitive decline *n* = 85 (16.2%)	No cognitive decline *n* = 441 (83.8%)	*P*-value
Female sex, %	58.8	57.6	0.83
Age, years	74.1 (6.3)	71.3 (5.4)	<0.01
≥13 years of education, %	2.5	15.2	<0.01
Living alone, %	21.2	22.7	0.75
Going outdoors more than once a day, %	85.9	94.1	<0.01
Chronic disease, %			
Hypertension	37.6	36.0	0.77
Hyperlipidemia	21.2	21.6	0.93
Cerebral vascular disease (stroke, cerebral hemorrhage, subarachnoid hemorrhage)	4.7	3.9	0.72
Heart disease (angina, myocardial infarction, arrhythmia, others)	12.9	11.1	0.63
Diabetes mellitus	7.1	10.9	0.28
Use of antihypertensive medication, %	34.1	33.3	0.89
BMI, kg/m^2^	23.3 (2.9)	23.3 (3.3)	0.99
Blood pressure, mm Hg			
Systolic	133 (20)	128 (18)	0.08
Diastolic	77 (11)	75 (11)	0.22
Pulse pressure	56 (15)	53 (13)	0.09
PWV, cm/s	1928 (379)	1754 (353)	<0.01
ABI	1.15 (0.06)	1.15 (0.06)	0.78
Grip strength, kg	25.0 (8.8)	26.1 (8.3)	0.24
Usual walking speed, m/sec	1.32 (0.25)	1.40 (0.21)	<0.01
Maximum walking speed, m/sec	1.86 (0.35)	1.98 (0.31)	<0.01
White blood cell count, /µL	5701 (1402)	5422 (1534)	<0.05
Red blood cell count, 10^4^/µL	444 (46)	443 (39)	0.67
Hemoglobin, g/dL	13.8 (1.5)	13.8 (1.2)	0.82
Hematocrit, %	41.9 (4.3)	41.6 (3.5)	0.76
Mean corpuscular volume, fL	94.5 (5.4)	94.0 (4.8)	0.79
Hemoglobin A1c, %	5.4 (0.6)	5.4 (0.7)	0.80
Total cholesterol, mg/dL	205 (34)	207 (34)	0.81
HDL cholesterol, mg/dL	57.0 (12.3)	59.2 (15.4)	0.29
Triglycerides, mg/dL	151 (79)	152 (79)	0.71
Creatinine, mg/dL	0.78 (0.16)	0.79 (0.19)	0.88
Albumin, g/dL	4.2 (0.3)	4.2 (0.3)	0.78
TMIG-IC full score, %	47.1	56.2	0.12
GDS Short-version ≥6 points, %	17.6	12.5	0.20
MMSE ≥26 points, %	94.1	85.9	<0.05
APOE genotype, %			0.87
ε2ε2	0	0.2	
ε2ε3	4.7	7.3	
ε2ε4	0	0.9	
ε3ε3	77.6	73.7	
ε3ε4	17.6	16.9	
ε4ε4	0	0.9	
Duration of follow-up, years	3.8 (0.8)	3.4 (1.9)	<0.01

ABI, ankle-brachial pressure index; APOE, apolipoprotein E; BMI, body mass index; GDS, Geriatric Depression Scale; HDL, high-density lipoprotein; MMSE, Mini-Mental State Examination; PWV, pulse wave velocity; TMIG-IC, Tokyo Metropolitan Institute of Gerontology Index of Competence.

Values are reported as percentage or mean (standard deviation).

**Figure 2.**
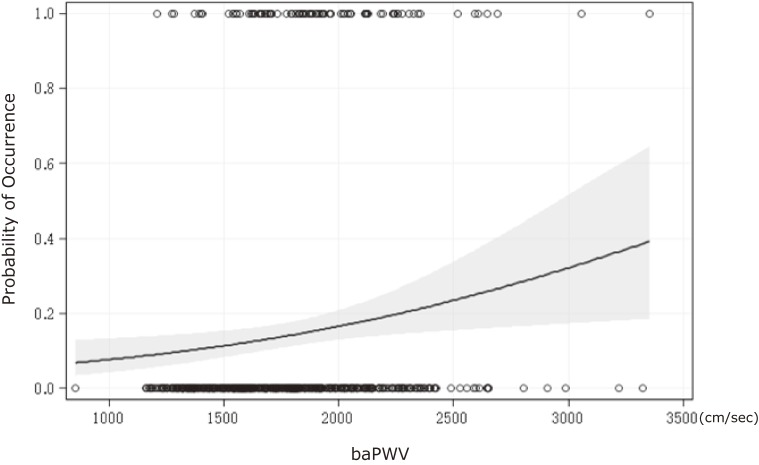
Logistic regression plot shows the relationship between brachial-ankle pulse wave velocity (baPWV) at baseline and the probability of occurrence for subsequent cognitive decline (solid line) adjusted for sex, age, and follow-up year, with 95% confidence intervals (gray) among community-dwelling Japanese aged ≥65 years.

Figure 2 clearly shows that the probability of CD occurrence increased with increasing baPWV, after controlling for sex, age, and duration of follow-up. Table 2 shows the associations of baPWV with CD, after controlling for potential confounders, in the four statistical models. Among the highest and middle tertiles of baPWV, the odds ratios (ORs) for CD were 3.88 (95% confidence interval [CI], 1.99–7.54) and 2.61 (95% CI, 1.32–5.18), respectively, compared with participants in the lowest tertile in the unadjusted model (model 1). After controlling for factors that were significantly associated with CD in univariate analysis (excluding maximum gait speed) (model 3), the corresponding ORs were 2.76 (95% CI, 1.30–5.84) and 2.35 (95% CI, 1.12–4.95). Furthermore, when potential confounders for CD were added to the model (model 4), baPWV remained independently significant: participants in the highest and middle tertiles of baPWV had ORs of 2.95 (95% CI, 1.29–6.74) and 2.39 (95% CI, 1.11–5.15), respectively, for CD.

**Table 2.** Independent associations of brachial-ankle pulse wave velocity at baseline with subsequent cognitive decline in community-dwelling Japanese aged ≥65 years

Independent variable	Model 1 (*n* = 524)	Model 2 (*n* = 524)	Model 3 (*n* = 487)	Model 4 (*n* = 483)
			
	OR (95% CI)	OR (95% CI)	OR (95% CI)	OR (95% CI)
PWV tertile				
lowest (reference)	1	1	1	1
middle	2.61 (1.32–5.18)**	2.44 (1.21–4.90)*	2.35 (1.12–4.95)*	2.39 (1.11–5.15)*
highest	3.88 (1.99–7.54)**	2.90 (1.44–5.86)**	2.76 (1.30–5.84)**	2.95 (1.29–6.74)**

Female sex		1.15 (0.74–1.88)	0.92 (0.54–1.57)	0.99 (0.57–1.73)
Age, per year		1.06 (1.02–1.11)*	1.04 (0.99–1.09)	1.05 (0.99–1.10)
Duration of follow-up, per year		1.51 (1.14–2.01)*	1.45 (1.05–2.00)*	1.55 (1.11–2.16)**

≥13 years of education			0.19 (0.04–0.82)*	0.20 (0.04–0.87)*
Going outdoors more than once a day			0.47 (0.21–1.05)	0.48 (0.22–1.09)
Usual walking speed, per tertile increase			0.81 (0.57–1.14)	0.81 (0.57–1.15)
White blood cell count, per tertile increase			1.23 (0.89–1.71)	1.17 (0.84–1.64)
MMSE ≥26 points			2.82 (1.03–7.69)*	2.78 (1.01–7.68)*

Use of antihypertensive medication				0.77 (0.43–1.38)
Systolic blood pressure, per tertile increase				1.06 (0.75–1.52)
HDL cholesterol, per tertile increase				0.82 (0.59–1.15)
Albumin, per tertile increase				1.00 (0.78–1.33)
APOE genotype, ε4				0.85 (0.42–1.72)

APOE, apolipoprotein E; CI, confidence interval; HDL, high-density lipoprotein; OR, odds ratio; PWV, pulse wave velocity; MMSE, Mini-Mental State Examination.

*P < 0.05, **P < 0.01.

Tertiles were defined as follows: usual walking speed (m/s): ≤1.29, 1.29–1.47, ≥1.47; white blood cell count (/µL): ≤4700, 4700–5800, ≥5800; systolic blood pressure (mm Hg): ≤121, 121–137, ≥137; HDL cholesterol (mg/dL): ≤51, 51–63, ≥63; albumin (g/dL): ≤4, 4–4.3, ≥4.3.

## DISCUSSION

Recent cross-sectional studies^5^^,^^14^ reported an association between baPWV and cognitive function; however, the longitudinal association with CD has not previously been studied. The present prospective study of community-dwelling older Japanese is the first to show that baPWV is an independent predictor of CD, as assessed by MMSE, after adjusting for potential confounders.

Studies have revealed several pathways between arterial stiffness and cognitive function. Most importantly, higher blood pressure level may have a key role in CD.^5^^,^^14^^,^^28^^–^^31^ In the present study, baPWV was independently associated with CD after adjustment for blood pressure level and use of antihypertensive medication. Furthermore, one study found that a baPWV value in the highest quartile was a significant predictor of progression to higher blood pressure categories,^32^ which suggests that arterial stiffness develops before hypertension.^13^^,^^33^

The present evidence suggests two mechanisms by which baPWV may predict CD development through higher blood pressure. First, blood flow disturbance may contribute to CD. Lower arterial elasticity leads to hypertension,^33^ which might accelerate narrowing of cerebral vasculature, leading to chronic hypoperfusion^8^ and ischemia. The resulting state of chronic hypoperfusion may impede delivery of energy substrates and nutrients to active brain cells and directly injure cerebral white matter or allow toxic metabolic byproducts to accumulate within the brain and blood vessels.^34^ In addition, ischemia may lead to accumulation of amyloid precursor protein and accelerated formation of free oxygen radicals, as has been seen in animal experiments.^35^ Second, certain diseases might have mediating roles in the pathophysiology of CD. Hypertension induced by arteriosclerosis may lead to thickening of the cerebrovascular endothelium and endothelial dysfunction and contribute to thrombosis and microinfarcts.^8^^,^^36^ Moreover, dysfunction in the blood-brain barrier could be important in CD. Concurrent breakdown of the blood-brain barrier may allow toxins, proteases, or other substances in the blood to enter the brain interstitial space and injure surrounding neurons and glial cells.^8^^,^^37^ Other studies have reported a significant positive relationship between arterial stiffness and volume or localization of white matter lesions, a known predisposing factor for dementia. A higher PWV was significantly associated with a greater volume of white matter hyperintensities on neuroimaging.^38^^–^^40^

The incidence rate of CD was 16.2% during a mean follow-up of 3.4 years. This value is compatible with previous results.^20^ In univariate analysis, CD was closely associated with age, years of education, frequency of going outdoors, usual and maximum gait speeds, WBC count, MMSE score, years of follow-up, and baPWV, and marginally associated with systolic blood pressure and pulse pressure. Andrew and colleagues^41^ found that increasing social vulnerability was associated with CD, and Saczynski and colleagues^42^ reported that a decline in social engagement between mid- and late-life was predictive of incident dementia. Further research is thus needed to explore the associations of social factors, such as frequency of going outdoors, with CD. Our findings support previous results on gait and cognitive function.^3^ Although a cross-sectional study found an inverse association between WBC count and psychomotor cognitive performance in the elderly,^43^ longitudinal associations have not been examined. Further study is necessary to examine the independent association of WBC count with CD, after controlling for cytokines and factors derived from leucocytes. In previous studies, the association between higher MMSE scores and CD was attributed to a ceiling effect.^3^^,^^4^ Several studies have shown that the ε4 allele is a risk factor for cognitive impairment and decline^44^^–^^46^; however, no studies found an effect of APOE genotype on cognitive functioning.^47^^–^^49^ The present findings are comparable to those of previous investigations, which indicates that our study population was not unusual.

This study has several strengths. First, we assessed a number of sociomedical factors and included them in statistical models, which revealed that baPWV was significantly associated with CD even after adjusting for sociodemographic factors, disease status, physical performance, nutritional status, social activities, and genotype. Second, PWV, as defined by the arterial radius and thickness of the arterial wall, can be used as a direct marker of arterial stiffness.^14^^,^^50^ We evaluated PWV using baPWV, which might be more relevant and practical in clinical settings, thus facilitating screening of persons at high risk.

Several limitations of this study should be noted. First, the participants were healthier than the general elderly population in Japan due to the “healthy volunteer effect”.^51^ A comparison of those followed-up (*n* = 526) and those excluded from the analysis (*n* = 456) showed that the latter had higher baPWV values and worse MMSE scores at baseline. This limitation may have attenuated the association between baPWV and CD. Second, cognition was assessed only with the MMSE. Although the MMSE is a convenient measure of global cognition, other instruments are available for such assessment. Further study is needed to explore the association of baPWV and other measures of CD (eg, by testing memory, psychomotor speed, and executive function).^52^

In conclusion, this prospective study found an association between higher baPWV and CD among community-dwelling older Japanese, even after adjusting for potential confounders. Stiffness in elastic arteries has been used as a marker of subsequent CD. Our findings suggest that stiffness in both muscular and elastic arteries is also a marker for subsequent CD, as it reflects disturbances in blood flow and/or the effects of certain diseases. These results may aid in risk assessment of elderly adults and might be useful in early screening for dementia.
